# Molecular Surveillance and Partial Genetic Characterization of Low-Pathogenic Avian Influenza A Viruses in Migrating Birds from Ukraine

**DOI:** 10.3390/v18101060

**Published:** 2026-09-24

**Authors:** Anton Gerilovych, Ivan Rusev, Ihor Korovin, Anastasiia Rusanova, Gregory E. Glass

**Affiliations:** 1 One Health Scientific and Research Institute, Private Scientific Institution, 61001 Kharkiv, Ukraine; 2 Institute for Problems of Cryobiology and Cryomedicine of NAS of Ukraine, 61015 Kharkiv, Ukraine; 3 National Nature Park “Tuzlivski Lymany”, 68100 Tatarbunary, Ukraine; rusevivan@ukr.net; 4 G&B Analytics, LLC, Gainesville, FL 32608, USA

**Keywords:** avian influenza virus, influenza A virus, migratory birds, Oxford Nanopore sequencing, genomic surveillance, phylogenetic analysis, H1, H4, H6, One Health, Ukraine

## Abstract

Wild migratory birds are the principal natural reservoir of influenza A viruses (IAVs) and play a central role in their evolution and global dissemination. Ukraine lies along the Black Sea–Mediterranean Flyway, making surveillance of avian influenza viruses in wild birds essential within a One Health framework, although genomic data from the region remain limited. Between October 2025 and January 2026, 604 cloacal and oropharyngeal swabs and environmental fecal samples were collected from migratory birds and mixed-species flocks in the Tuzlivski lymany National Nature Park and surrounding areas, together with samples from nearby domestic poultry. Influenza A virus was detected by real-time RT-PCR targeting the matrix gene, followed by H5/H7 screening, Oxford Nanopore sequencing of complete hemagglutinin genes, and phylogenetic analysis. Influenza A virus RNA was identified in 30/604 samples (4.97%), whereas all samples were negative for H5 and H7 subtypes. Complete hemagglutinin sequences revealed three low-pathogenic subtypes (H1, H4, and H6) that clustered within established Eurasian lineages closely related to contemporary avian viruses circulating across Europe, Asia, and North America. No influenza A virus or Newcastle disease virus was detected in domestic poultry. These findings provide an important genomic baseline for avian influenza surveillance in Ukraine and demonstrate the value of portable nanopore sequencing and integrated One Health surveillance for monitoring viral diversity and supporting early detection of emerging influenza threats.

## 1. Introduction

Avian influenza virus (AIV) remains one of the most important emerging pathogens affecting animal health, wildlife conservation, and global public health. Influenza A viruses belong to the species *Alphainfluenzavirus influenzae* from genus *Alphainfluenzavirus* family Orthomyxoviridae and are classified according to the antigenic properties of their hemagglutinin (HA; H1–H16, and H19 in birds, and H17–H18 in bats) and neuraminidase (NA; N1–N11) glycoproteins. Wild aquatic birds of the orders *Anseriformes* and *Charadriiformes* constitute the natural reservoirs for the majority of influenza A virus subtypes, maintaining viral diversity through extensive migration and intercontinental dissemination [1,2,3]. Most viruses circulating in these hosts are classified as low pathogenic avian influenza (LPAI) viruses; however, their continued evolution, reassortment, and occasional spillover into domestic poultry and mammals create opportunities for the emergence of highly pathogenic strains with significant veterinary and zoonotic consequences [4,5].

The movement of migratory birds along major flyways plays a central role in the global ecology of avian influenza viruses. The Black Sea–Mediterranean Flyway is one of the most important migratory routes connecting Eurasia, Africa, and Western Asia, enabling seasonal exchange of waterfowl and shorebird species that facilitate virus maintenance and long-distance transmission [6]. Ukraine is in a strategically important geographic position within this flyway. It is a major stopover and wintering area for millions of migratory birds. Wetland ecosystems along the Black Sea coast, including the Tuzly Estuaries National Nature Park, provide ideal ecological conditions for interactions among migratory waterfowl, resident wild birds, and domestic poultry, thereby increasing opportunities for virus transmission across species [7].

Although highly pathogenic H5 clade 2.3.4.4 viruses have caused substantial outbreaks in Europe over the last decade, surveillance studies consistently demonstrate that low-pathogenic influenza viruses circulate are endemic in wild aquatic bird populations and serve as the principal genetic reservoir from which novel reassortants may emerge [8,9]. Consequently, continuous surveillance of apparently healthy migratory birds is considered an essential component of early warning systems for emerging influenza viruses under the One Health framework, integrating animal, human, and environmental health [10]. Additionally, surveillance of nearby domestic animals provides evidence of early spillover into human-associated species, as well as answering the conceptual question of how often these spillover events occur.

Traditional surveillance of avian influenza relies primarily on virus isolation and real-time reverse transcription polymerase chain reaction (RT-qPCR), which provide sensitive detection but limited information of viral genetic diversity and evolutionary relationships [11]. Advances in next-generation sequencing (NGS), particularly portable Oxford Nanopore Technologies platforms, have expanded opportunities to rapidly characterize genomes directly from field-derived samples. Long-read sequencing enables recovery of complete influenza gene segments, facilitates subtype identification, detects genetic reassortment, and supports near-real-time molecular epidemiology in both laboratory and field settings [12,13]. These technologies have become increasingly valuable for genomic surveillance of emerging viral pathogens because they provide rapid turnaround times, portability, and relatively low infrastructure requirements.

The hemagglutinin (HA) gene is of particular epidemiological importance because it determines viral receptor specificity, host adaptation, antigenicity, and pathogenicity. Phylogenetic analysis of HA sequences allows reconstruction of viral evolutionary history, identification of transmission pathways, and comparison of local isolates with globally circulating lineages [1,13]. Molecular characterization of HA genes therefore provides critical information for understanding the ecology of influenza viruses circulating in migratory birds and contributes to international surveillance databases.

Despite Ukraine’s critical geographic positioning within the intersection of major Eurasian migratory waterfowl flyways, comprehensive genomic data describing avian influenza viruses (AIVs) circulating within local wild bird populations remains limited [7]. Historical surveillance networks within the region have predominantly focused on the target detection of high-consequence outbreaks, such as those caused by highly pathogenic avian influenza (HPAI) H5 variant lineages [14]. Consequently, molecular and evolutionary data regarding low-pathogenic avian influenza (LPAI) strains silently maintained in natural reservoirs have been comparatively scarce [15,16]. Expanding continuous genomic and molecular surveillance programs is therefore vital to improve regional biopreparedness, elucidate shifting viral evolutionary dynamics, and strengthen international early warning frameworks [17,18,19].

This study investigated the circulation of Influenza A viruses among wild and domestic birds inhabiting the Tuzlivski Lymany National Nature Park and surrounding settlements in Southern Ukraine using a One Health surveillance approach. Specifically, we combined RT-qPCR screening, Oxford Nanopore sequencing of complete HA gene segments and phylogenetic analyses to identify circulating influenza A virus subtypes and characterize their genetic relationships with strains, isolated worldwide, and evaluated their genetic homology with contemporary Eurasian and America avian and swine influenza viruses.

As part of the implementation of the scientific cooperation agreement between the Tuzlivski lymany National Nature Park (NNP TL) and the Private Scientific Institution “Research Institute of One Health” (OHSRI, PSI), field studies were conducted during autumn and winter in 2025–2026 within the NPP TL and adjacent settlements to investigate the circulation of orthoviruses and paramyxoviruses among wild and domestic birds in the context of implementing the “One Health” approach.

The goal of the research was to establish a baseline and study the potential circulation of pathogens in wild bird populations and to monitor their possible transmission to domestic bird populations.

## 2. Materials and Methods

Sampling. Sampling was carried out between October 2025 and January 2026 within the Tuzlivski lymany National Nature Park and in neighboring settlements. Both wild and domestic birds were sampled.

Samples from wild birds were obtained in two main ways. First, individual birds were netted. Captured individuals were identified to species, sex was determined (when possible), morphometric measurements were taken, and individual data were recorded. Biological samples (cloacal and oropharyngeal swabs) were then collected, and the birds were released back into the wild. Some individuals were recaptured, which was recorded in the logbooks (Table 1).

Secondly, wild bird droppings were collected on sandbars, in shallow waters and in coastal sections of estuaries within the preserve. Samples were collected from fresh droppings, often from mixed flocks of waterfowl and shorebirds (ducks, waders, herons, etc.), without individual identification of each bird (Table 2).

Samples from domestic birds (chickens, geese, Indian runner ducks) were collected from private households in settlements located adjacent to the park. As with netted wild birds, cloacal swabs were collected, and droppings were obtained. These domestic birds included semi-free-range rearing (for example, ducks that spend the day in natural water bodies and the night on farms) (Table 3). These rural households did not maintain any biosecurity conditions to prevent contact with wild birds.

All samples were labelled, with the collection site and date recorded, and then transported to the laboratory for further analysis using molecular genetic methods. This approach ensured coverage of both wild bird populations and domestic birds in contact with the natural environment and allowed for an assessment of their potential role in the circulation of infectious agents.

Sample preparation. Clinical (cloacal and pharyngeal swabs) and environmental (wild birds’ feces) samples to be tested for Influenza A virus were stored in viral transport media (VTM) at −80 °C until processing. Viral RNA was extracted from 150 μL of each sample using the MagnetPure DNA/RNA Kit (IBT, Kyiv, Ukraine) according to the manufacturer’s instructions. RNA was eluted in 80 μL of nuclease-free water and immediately used for reverse transcription and screening PCR.

PCR screening. Screening amplification was performed under E. Spackman protocol [10] with determination of presence of Influenza A virus RNA and confirmation of presence or absence of H5 and H7 subtypes of virus. In addition, all samples were screened for the presence of Newcastle disease virus using a pooling method [20].

Multi-segment RT-PCR amplification of the HA gene. To amplify the full-length Hemagglutinin (HA) gene segment (~1.7 kb), a universal multi-segment RT-PCR approach was adapted from the Hoffmann et al. (2001) protocol [21]. A one-step RT-PCR was performed using the EvaGreen based Solis Biodine Kit (Tartu, Estonia).

The universal primer cocktail targeted the highly conserved non-coding terminal regions of the Influenza A genome: Modified universal primers (5’-ACGCGTGATCAGCAAAAGCAGG-3’ and 5’-ACGCGTGATCAGTAGAAACAAGG-3’) were used for amplification of viral genetic material.

The 50 μL reaction mixture contained 25 μL of 2× Platinum SuperScript IV RT-PCR Master Mix, 0.5 μL of SuperScript IV RT-PCR Enzyme Mix, 0.4 μM of each primer, RNA-extract: 5–10 μL, with concentration 10 ng/μL, and nuclease-free water to final volume [21].

The thermal cycling profile was executed on iTower RT-PCR-machine (Jena Analytic, Jena, Germany) with the following parameters: reverse Transcription: 50 °C for 20 min, initial Denaturation: 95 °C for 2 min, amplification 35 cycles: denaturation: 98 °C for 10 s, annealing: 55 °C for 30 s, extension: 72 °C for 90 s, and final extension: 72 °C for 5 min, followed by a hold at 4 °C.

PCR products were visualized via 1% agarose gel electrophoresis to confirm the presence of the ~1.7 kb band of HA segment. Amplicons were purified using Agencourt AMPure XP beads (Beckman Coulter, Brea, CA, USA) and quantified using a Qubit 4.0 Fluorometer with the Qubit 1× dsDNA HS Assay Kit (Thermo Fisher Scientific, San Diego, CA, USA) [22,23].

NGS Library Preparation and Sequencing. Native barcoding of full-length unfragmented HA amplicons was performed using the ONT Native Barcoding Kit (SQK-NBD114.24, Didcot, UK). A total of 400 ng of purified PCR product per sample was used for end-prep and adapter ligation. Barcoded samples were pooled in equal ratios [24].

The final sequencing library was loaded onto a R10.4.1 Flow Cell (FLO-PRO114M) and sequenced on a MinION Mk1B device using MinKNOW software v. 25.03.9 with super-accuracy basecalling enabled.

Bioinformatics and data analysis. Raw sequencing reads underwent quality control filtering and adapter trimming using Porechop for ONT [25]. Low-quality reads (Q < 10) and reads shorter than 100 bp were excluded. High-quality reads were analyzed with BLASTn comparison, and after recognition of belonging to H1, H4, and H6 subtypes were mapped against reference sequences matching those subtypes retrieved from the NCBI Influenza Virus Database or GISAID using Minimap2. Consensus sequences were generated using by Medaka with a minimum coverage depth threshold of 100×. Subtypes were verified in silico using the FluSurver tool (GISAID) and NCBI Influenza Virus Sequence Annotation Tool [26,27].

Phylogenetic Analysis. Consensus HA gene sequences were aligned alongside global reference strains using MAFFT (v7). Phylogenetic trees were reconstructed using the Maximum Likelihood method in MEGA12, applying the best-fit nucleotide substitution model determined by ModelFinder. The sequences of each defined subtypes were retrieved from NCBI Virus database (https://www.ncbi.nlm.nih.gov/genomes/FLU/Database/nph-select.cgi?go=database, accessed 21 September 2026) and used for phylogenetic analysis.

Tree topology robustness was evaluated with 1000 bootstrap replicates [28].

Sequences were deposited in GenBank using BankIT system for Influenza viruses.

## 3. Results

Detection of Influenza A virus and subtyping. All collected clinical (cloacal and oropharyngeal swabs) and environmental (wild bird fecal) samples were initially screened by real-time RT-PCR targeting the matrix (M) gene of Influenza A virus. Influenza A virus RNA was detected in selected samples, which were then analyzed for H5 and H7 subtypes. None of the Influenza A-positive samples were H5 or H7, indicating the circulation of other avian influenza virus subtypes among the investigated wild bird populations.

The presence of influenza A virus RNA not belonging to high-risk or highly pathogenic subtypes was detected in 5.2% of clinical samples and 2.15% of wild bird fecal samples (Table 1 and Table 2). This indicates the circulation of influenza A virus, typical for wild waterfowl and semi-aquatic bird species. The percentage of positive samples does not exceed the regional average for previous years (14, 15, personal observations).

No influenza A virus or Newcastle disease virus RNA was detected in samples from domestic poultry (cloacal swabs and droppings), indicating that the households studied were free from the target infections (Table 3). Despite the close proximity to infected wild birds and the lack of biosecurity measures, spillover was not detected.

In addition, over 600 samples of clinical materials were collected for the study, including cloacal swabs from individual birds and environmental samples (droppings from mixed flocks).

The vast majority of samples were obtained from waterfowl, particularly members of the order *Anseriformes*. The dominant species was the mallard (*Anas platyrhynchos*), which constituted the largest proportion of the sample (n ≈ 187). A significant number of samples were also obtained from the white-fronted goose (*Anser albifrons*, n = 161) and the whooper swan (*Cygnus cygnus*, n = 50).

Among the representatives of the order *Charadriiformes*, *Calidris alpina*, n > 90 was the most numerous. Other species, including *Recurvirostra avosetta*, *Tringa nebularia*, and *Pluvialis squatarola,* were represented by single samples.

In addition, fecal samples were collected from ecologically significant species such as the black-necked grebe (*Podiceps nigricollis*, n = 12), and from mixed flocks of herons (*Egretta alba*, *Ardea cinerea*) and ducks (*Anas* spp.), yielding another 100 samples.

The results of testing for NDV of all samples were negative. The results concerning Influenza A virus genetic material are presented in the tables below (Table 4, Table 5 and Table 6).

A total of 604 samples were collected from wild birds and environmental mixed-species flocks in the National Nature Park “Tuzlivski Lymany” and adjacent areas between November 2025 and January 2026 and tested. Of these, 504 samples were attributed to individual bird species, while 100 samples originated from mixed-species flocks.

Screening for Influenza A virus (IAV) detected 30 positive samples (4.97%). No samples tested positive for highly pathogenic subtypes either the H5 or H7 avian influenza virus subtypes.

Among individually identified species, IAV RNA was detected in 27 of 504 samples (5.36%). The highest numbers of positive samples were recorded in Mallards (*Anas platyrhynchos*), with 12 positive samples out of 108 tested (11.1%), followed by Greater white-fronted geese (*Anser albifrons*), with 8 positive samples out of 161 tested (5.0%), and Dunlins (*Calidris alpina*), with 4 positive samples out of 98 tested (4.1%). Single positive samples were identified in Whooper swan (*Cygnus cygnus*), Mute swan (*Cygnus olor*), and Black-necked grebe (*Podiceps nigricollis*).

Among the mixed-species samples, 3 of 100 samples (3.0%) were positive for IAV, including 2 positive fecal samples collected from a mixed flock of Eurasian wigeons (*Anas penelope*) and Eurasian teal (*Anas crecca*), and 1 positive sample from a mixed flock of Great egrets (*Ardea alba*) and Grey herons (*Ardea cinerea*).

Overall, the results indicate ongoing circulation of low-pathogenic Influenza A viruses among multiple wild bird species inhabiting the Black Sea coastal wetlands of Ukraine during the autumn–winter migration period of 2025. The absence of H5 and H7 subtypes provides no evidence of highly pathogenic avian influenza viruses in the sampled populations at the time of surveillance.

Amplification and Nanopore Sequencing of the HA Gene. The universal multi-segment RT-PCR protocol successfully amplified the complete hemagglutinin (HA) gene segment (~1.7 kb) from selected Influenza A-positive samples. Agarose gel electrophoresis demonstrated distinct amplicons of the expected size with minimal nonspecific amplification.

Following purification and native barcoding, all libraries generated sufficient sequencing output on the Oxford Nanopore MinION platform. Quality filtering removed low-quality reads and adapter sequences, while the remaining reads provided high genome coverage for consensus sequence generation. Mean sequence coverage exceeded the threshold required for reliable consensus reconstruction (>100×), allowing accurate subtype determination.

Identification of Influenza A virus subtypes. Bioinformatic analysis confirmed three hemagglutinin subtypes among the analyzed samples: H1, H4, and H6. Subtype assignments obtained through reference mapping were independently confirmed using FluSurver and the NCBI Influenza Virus Sequence Annotation Tool.

Consensus HA sequences were submitted to GenBank under the following accession numbers: H1: PZ558332.1, PZ558541.1, PZ558585.1; H4: PZ559198.1, PZ558750.1; H6: PZ558586.1. These sequences represent recent Ukrainian avian influenza virus isolates and expand the publicly available genetic data for circulating low-pathogenic avian influenza viruses.

Phylogenetic Analysis. Maximum Likelihood phylogenetic analysis of the complete HA gene demonstrated that the Ukrainian isolates clustered in their respective HA subtypes (Figure 1, Figure 2 and Figure 3).

The generated HA sequences provide valuable baseline data for molecular surveillance and contribute to the understanding of avian influenza virus evolution and geographic spread within the Eurasian flyway.

Maximum Likelihood phylogenetic analysis of the complete HA gene demonstrated that the three Ukrainian H1 isolates clustered within the *Alphainfluenzavirus influenzae* H1 viruses from Germany, Belgium (2017), and Canada (2010). The isolates A/Calidris alpina/Ukraine/06-2025/2025(H1) (PZ558585.1), A/Anas platyrhynchos/Ukraine/03-2025/2025(H1) (PZ558541.1), and A/Anas platyrhynchos/Ukraine/02-2025/2025(H1) (PZ558332.1) formed a closely related monophyletic cluster, indicating limited genetic divergence among H1 subtype viruses circulating among wild birds in sampling area in Ukraine (Figure 1).

Also, the online BLASTn (https://blast.ncbi.nlm.nih.gov/Blast.cgi?PROGRAM=blastn&PAGE_TYPE=BlastSearch&LINK_LOC=blasthome, accessed 21 September 2026) Ukrainian H1 viruses are closely grouped with recent H1 viruses isolated from domestic and wild pigs. Their placement within a well-supported clade of low-pathogenic avian influenza viruses suggests that these isolates belong to globally circulating avian/swine H1 gene pools could be potentially presented in migratory bird populations. No association with highly pathogenic influenza viruses was observed.

The isolates A/Philomachus pugnax/Ukraine/05-2025/2025(H4) (PZ559198.1) and A/Tringa totanus/Ukraine/04-2025/2025(H4) (PZ558750.1) were clustered with H4 viruses previously detected in wild ducks, mallards, geese, and other aquatic birds from Europe and Asia, including isolates from China and Taiwan. The Ukrainian virus occupied a terminal branch within this lineage without forming a distinct divergent cluster, indicating high genetic similarity to contemporary wild bird H4 viruses.

The overall topology demonstrates that the Ukrainian H4 virus is part of the established migratory bird transmission network and shares a recent common ancestor with other Eurasian low-pathogenic H4 viruses circulating along major migratory flyways.

The HA phylogeny of H6 viruses showed that the Ukrainian isolate A/Calidris alpina/Ukraine/06-2025/2025(H6) (PZ558586.1) clustered within the Asian (Viet Nam and China) wild bird H6 lineage (Figure 3).

The Ukrainian H6 virus did not occupy a basal or highly divergent position but instead grouped within a contemporary cluster of low-pathogenic H6 viruses, suggesting recent introduction through natural wild bird migration rather than long-term independent evolution.

Overall phylogenetic relationships. The phylogenetic analyses consistently demonstrated that all Ukrainian isolates belonged to established low-pathogenic avian influenza virus lineages maintained in wild bird reservoirs. Despite representing three distinct HA subtypes (H1, H4, and H6), all viruses clustered with contemporary isolates from migratory waterfowl and shorebirds, highlighting the important role of migratory birds in the introduction and dissemination of influenza A viruses into Ukraine.

Instead, the Ukrainian viruses showed close evolutionary relationships with avian viruses circulating across Eurasia and North America, consistent with ongoing gene flow along international migratory flyways. Also, the relations of H1 subtype detected viruses with swine originated European isolates were noticed.

These findings indicate that the Ukrainian isolates are representative of the global avian and swine influenza virus gene pool and provide valuable baseline genomic data for molecular surveillance of wild bird influenza viruses in Eastern Europe.

## 4. Discussion

The present study provides new evidence for the widespread circulation of low-pathogenic avian influenza A viruses (LPAIV) among migratory wild birds in the Black Sea coastal wetlands of Ukraine. By combining molecular screening, Oxford Nanopore sequencing, and phylogenetic analysis, we detected influenza A viruses belonging to the H1, H4, and H6 hemagglutinin subtypes were moderately common, while there was an absence of detection of highly pathogenic H5 and H7 viruses in sampled clinically healthy birds during the period of testing the autumn and winter of 2025–2026. It is important to emphasize that the non-detection of H5 and H7 subtypes in this study does not imply their total absence from the region. While the sample size was robust, the surveillance period was relatively brief. Therefore, our findings indicate only that H5 and H7 subtypes were below detectable levels or absent within the specific populations during the active sampling window. Given the transient nature of avian influenza dynamics in wild reservoirs, circulation outside this timeframe remains possible. These findings contribute important baseline genomic data for Ukraine and support the role of migratory waterbirds as natural reservoirs maintaining influenza A viruses of swine and bird origin in wild birds’ populations, and its diversity within the Eurasian flyway. The study also shows that despite the typical levels of LPAI circulating in wild birds, spillover into nearby domestic flocks was undetected.

Overall Influenza A virus positivity was approximately 5%, which is consistent with surveillance studies conducted across Europe and Asia that report prevalence rates ranging from 2% to 10% in apparently healthy migratory waterfowl, depending on season, sampling strategy, and host species [2,6]. The highest detection rates observed in mallards (*Anas platyrhynchos*) and greater white-fronted geese (*Anser albifrons*) are unsurprising because dabbling ducks are recognized as the principal natural hosts of influenza A viruses and frequently exhibit asymptomatic infections while shedding substantial quantities of virus into aquatic environments [1,13]. Similarly, the detection of influenza virus RNA in dunlins (*Calidris alpina*) confirms that shorebirds also participate in maintaining influenza virus diversity, particularly along coastal migratory routes.

The absence of H5 and H7 viruses is important from both veterinary and epidemiological perspectives. Since 2020, Europe has experienced unprecedented outbreaks of highly pathogenic H5N1 clade 2.3.4.4b viruses affecting wild birds and poultry, with spillover into numerous mammalian species [5,7,19]. Although no H5 or H7 viruses were identified during this study, the continued circulation of low-pathogenic influenza viruses should not be underestimated. LPAIV strains constitute the evolutionary reservoir from which highly pathogenic viruses emerge following mutation or reassortment, particularly after introduction into domestic poultry populations [3]. Therefore, continued surveillance remains essential even in the absence of highly pathogenic virus detection.

The identification of H1, H4, and H6 subtypes agrees well with previous surveillance conducted in migratory waterfowl throughout Eurasia. These subtypes are among the most frequently detected low-pathogenic influenza viruses in wild birds and have demonstrated broad geographic distribution across Europe, Asia, and North America [19,20,29,30]. H4 and H6 viruses are particularly common in aquatic bird populations and have occasionally contributed gene segments to reassorting viruses infecting poultry. Although H1 viruses are generally associated with low pathogenicity in birds, they represent an important component of the global influenza gene pool because reassortment between avian and mammalian influenza viruses may facilitate emergence of novel strains with altered host range [1].

Phylogenetic analyses demonstrated that all Ukrainian isolates clustered within established Eurasian avian influenza virus lineages and exhibited close genetic relationships with viruses recently detected in migratory birds from Asia, Europe, and genetic relations with North American isolates of 2000s and 2010s. This observation supports the hypothesis that influenza A viruses circulate continuously through interconnected migratory flyways rather than evolving independently within isolated geographic regions [6]. The relatively low divergence of sequences among the Ukrainian isolates further suggests recent introduction through seasonal migration rather than prolonged local evolution. Similar phylogeographic patterns have been described in multiple studies demonstrating regular intercontinental exchange of avian influenza viruses mediated by migratory birds [2,5].

An important strength of this study is the application of Oxford Nanopore sequencing for molecular surveillance. Portable long-read sequencing platforms have transformed infectious disease surveillance by enabling rapid sequencing directly from PCR products with minimal laboratory infrastructure [12]. The successful recovery of complete HA gene sequences from all selected positive samples demonstrates that nanopore sequencing is a practical approach for routine avian influenza surveillance in Ukraine. Although short-read sequencing technologies continue to provide greater per-base accuracy, improvements in nanopore chemistry and consensus algorithms have substantially increased sequence quality, making this technology suitable for phylogenetic investigations and outbreak response [11]. Furthermore, the ability to generate genomic data in near real time offers considerable advantages for monitoring emerging pathogens within the One Health framework.

The One Health design of this surveillance also provides valuable ecological information. Sampling included both wild migratory birds and domestic poultry located adjacent to protected wetland ecosystems. No influenza A virus RNA or Newcastle disease virus RNA was detected in domestic poultry, suggesting that, during the study period, virus circulation was not confirmed in domestic poultry. This is in a region under current geopolitical conflict, where resources for biosecurity and monitoring are extremely limited.

During the surveillance of semi-wild teals, a pooled sample initially demonstrated a weak positive signal for Influenza A virus RNA (Ct < 35). However, subsequent individual deconvolution of the pool yielded negative results (Ct less than 38 or No Ct). This discrepancy is a recognized limitation of pooling strategies in molecular diagnostics, often attributed to the dilution effect. When an individual sample with a very low viral load is pooled with negative samples, the target RNA concentration can be diluted near or below the assay’s analytical limit of detection (LoD). Consequently, while the aggregated target volume in the pool was sufficient to trigger a weak amplification signal, individual aliquots lacked the minimum template copy number required for reproducible detection under standard single-reaction parameters.

Nevertheless, the close proximity between free-ranging domestic birds and migratory waterfowl observed around the Tuzlivski Lymany creates opportunities for future spillover events. Similar wildlife–livestock interfaces have been identified as important epidemiological hotspots for influenza virus transmission worldwide [7,8]. Continued integrated surveillance of wildlife, domestic animals, and environmental samples is therefore essential for early detection of viral emergence.

Several limitations should be acknowledged. First, surveillance was conducted during a single migratory season and therefore does not fully capture seasonal or annual fluctuations in virus prevalence. Second, only the HA gene was sequenced, preventing comprehensive assessment of genome-wide reassortment or evolutionary dynamics involving the remaining seven influenza virus gene segments. Whole-genome sequencing would allow identification of reassortment events and provide greater insight into virus evolution. Finally, virus isolation was not performed for all positive samples, limiting opportunities for antigenic characterization and pathogenicity assessment.

Future studies should expand surveillance across multiple years and geographic regions of Ukraine while incorporating whole-genome sequencing and ecological metadata from host species. Integration of genomic surveillance with bird migration data, environmental monitoring, and international sequence databases would substantially improve understanding of influenza virus movement along the Black Sea–Mediterranean Flyway. Such efforts are particularly important given the increasing frequency of highly pathogenic avian influenza outbreaks globally and the ecological changes affecting migratory bird populations.

In conclusion, this study provides the first 2025/2026 molecular characterization of hemagglutinine gene of H1, H4, and H6 avian influenza viruses circulating among migratory birds in the Tuzlivski lymany National Nature Park using Oxford Nanopore amplicon-based sequencing. The detected viruses are related genetically with avian influenza viruses and swine-originated viruses of H1 subtype and exhibit close evolutionary relationships with contemporary viruses circulating in migratory birds. These findings strengthen baseline knowledge of avian influenza ecology in Ukraine and demonstrate the value of integrating molecular diagnostics, genomic surveillance, and phylogenetic analysis within a One Health framework for the early detection and monitoring of emerging influenza viruses.

## 5. Conclusions

This study demonstrates the successful application of an integrated molecular surveillance approach combining real-time RT-PCR, Oxford Nanopore next-generation sequencing, and phylogenetic analysis to investigate the circulation of avian influenza viruses in migratory birds inhabiting the Black Sea coastal wetlands of Ukraine. The findings expand current knowledge of influenza A virus ecology in Eastern Europe and provide valuable baseline genomic data for future surveillance.

The principal conclusions of the study are as follows:

1. Active circulation of low-pathogenic avian influenza viruses was confirmed among migratory wild birds in the Tuzlivski lymany National Nature Park, with an overall Influenza A virus positivity of tested samples of 4.97% (30/604 samples). No highly pathogenic H5 or H7 viruses were detected during the surveillance period, indicating that the circulating viruses belonged to low-pathogenic avian influenza lineages.
2. Wild waterfowl and shorebirds served as the primary natural reservoirs of influenza A viruses in Ukraine. The highest detection rates were observed in mallards (*Anas platyrhynchos*), greater white-fronted geese (*Anser albifrons*), and dunlins (*Calidris alpina*), highlighting their roles in maintaining and disseminating influenza viruses along the Eurasian migratory flyways.
3. Oxford Nanopore sequencing proved to be an effective tool for rapid genomic surveillance. Complete HA gene sequences were successfully generated from representative positive samples, allowing accurate subtype identification and high-confidence phylogenetic analyses while demonstrating the suitability of portable sequencing technologies for routine wildlife surveillance.
4. Three hemagglutinin subtypes (H1, H4, and H6) were identified, representing low-pathogenic avian influenza viruses commonly associated with migratory waterbirds. The obtained sequences were deposited in GenBank, providing new publicly available genomic resources describing contemporary Ukrainian influenza A virus isolates.
5. Phylogenetic analyses demonstrated that the Ukrainian isolates belong to established Eurasian avian influenza virus lineages. Most characterized viruses showed close evolutionary relationships with recent bird-originated isolates from Europe and Asia, supporting the hypothesis that migratory birds facilitate continuous intercontinental exchange of influenza A viruses through overlapping flyways.
6. The study establishes an important molecular baseline for Ukraine, providing reference genomic data that will support future monitoring of viral evolution, detection of emerging variants, and early warning systems for highly pathogenic avian influenza.

## Figures and Tables

**Figure 1:**
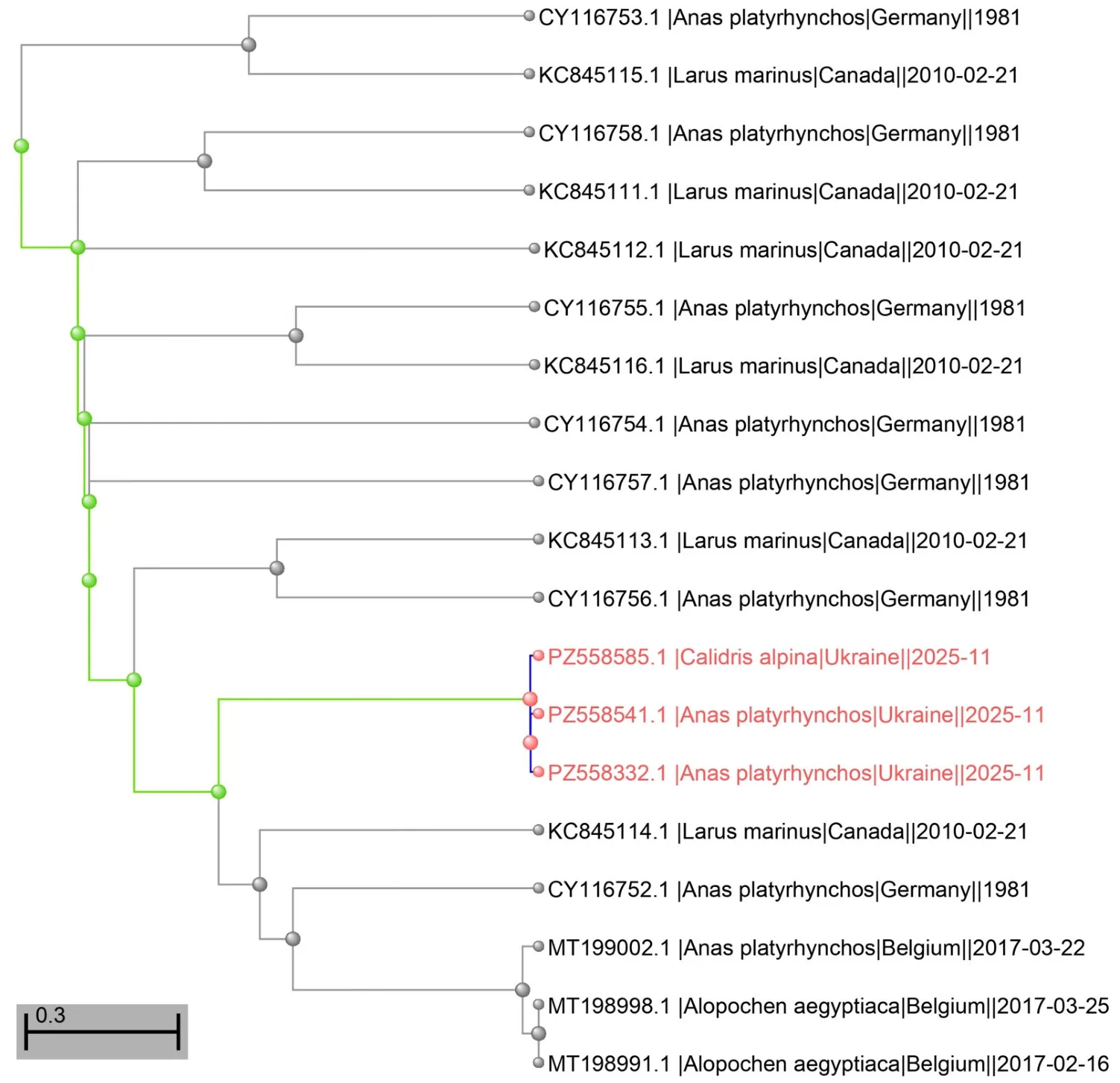
Phylogenetic relations of Ukrainian H1 *Alphainfluenzavirus influenzae* isolates by HA gene full length sequences (marked with red font). Phylogenetic analysis performed by MEGA v.12, method ML, Bootstrap rate 1000, applying the GTR nucleotide substitution model. Gaps and missing data were treated using partial deletion with a 95% site coverage cutoff.

**Figure 2:**
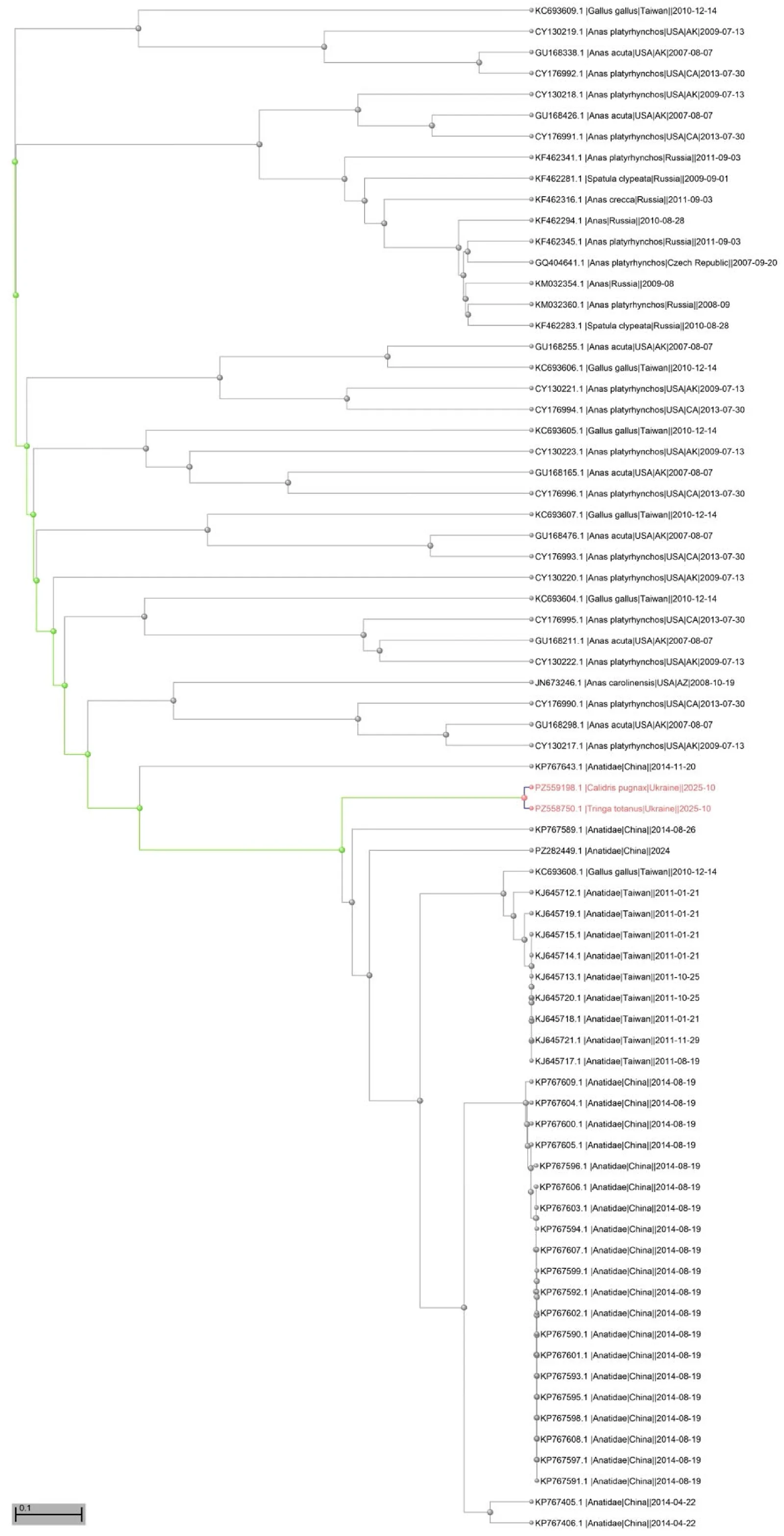
Phylogenetic relations of Ukrainian H4 *Alphainfluenzavirus influenzae* isolates by HA gene full length sequence (marked with red font). Phylogenetic analysis performed by MEGA v.12, method ML, Bootstrap rate 1000, applying the GTR nucleotide substitution model. Gaps and missing data were treated using partial deletion with a 95% site coverage cutoff.

**Figure 3:**
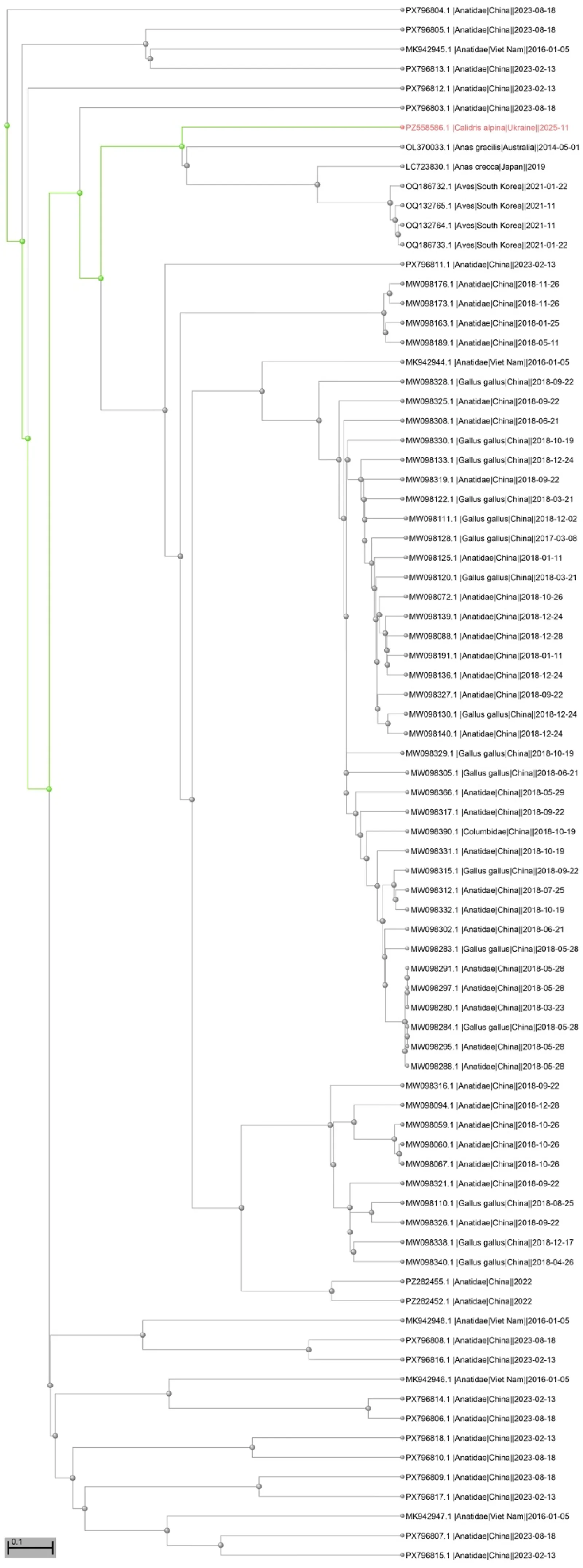
Phylogenetic relations of Ukrainian H6 *Alphainfluenzavirus influenzae* isolate by HA gene full length sequences (marked with red font). Phylogenetic analysis performed by MEGA v.12, method ML, Bootstrap rate 1000, applying the GTR nucleotide substitution model. Gaps and missing data were treated using partial deletion with a 95% site coverage cutoff.

**Table 1:** Results of testing clinical samples from wild birds (October–November 2025) for the presence of *Influenza A* and ND viruses.

Species	Species, Latinum	Quantity	Influenza A Virus	H5	H7	NDV
Mallard	*Anas platyrhynchos*	20	2	-	-	-
Eurasian teal	*Anas crecca*	2	-	-	-	-
Northern shoveler	*Anas clypeata*	1	-	-	-	-
Dunlin	*Calidris alpina*	72	2	-	-	-
Little stint	*Calidris minuta*	2	-	-	-	-
Common redshank	*Tringa totanus*	6	1	-	-	-
Common greenshank	*Tringa nebularia*	1	-	-	-	-
Ruff	*Philomachus pugnax*	5	1	-	-	-
Black-headed gull	*Chroicocephalus ridibundus*	1	-	-	-	-
Short-eared owl	*Asio flammeus*	1	-	-	-	-
Common reed bunting	*Emberiza schoeniclus*	1	-	-	-	-
European robin	*Erithacus rubecula*	1	-	-	-	-
Common snipe	*Gallinago gallinago*	1	-	-	-	-
Total		114	6	0	0	0

**Table 2:** Results of fecal samples from wild birds (October–November 2025) for the presence of *Influenza A* and ND viruses.

Group	Species or Genus in Latina	Quantity	Influenza A Virus	H5	H7	NDV
Mixed waders	*Recurvirostra avosetta*, *Philomachus pugnax*	71	2	-	-	-
Mixed waterbirds	*Anas*, *Tadorna*, *Ardea*, *Numenius*, *Tringa*	161	3	-	-	-
Total		232	5	0	0	0

**Table 3:** Results of tests on cloacal swabs and feces from domestic poultry (October–November 2025) for the presence of *Influenza A virus* and NDV.

Species	Samples Quantity	Influenza A Virus	H5	H7	NDV
Crossbred semi-wild	28	- *	-	-	-
Mallards	26	-	-	-	-
Chicken	13	-	-	-	-
Domestic geese	34	-	-	-	-
Domestic ducks	21	-	-	-	-
Total	122	0	0	0	0

Note: *—During the analysis of a sample pool from semi-wild teals, a weak signal was detected (Ct less than 35), indicating the presence of *Influenza A virus* RNA in the samples; however, upon individual testing, all samples proved negative (Ct less that 38 or No Ct).

**Table 4:** Aggregated bird samples by species, tested for IAV (November 2025–January 2026).

Species Name	Species in Latina	Quantity	IAV	H5	H7
Dunlin	*Calidris alpina*	98	4	-	-
Mallard	*Anas platyrhynchos*	108	12	-	-
Eurasian teal	*Anas crecca*	6	-	-	-
Greater white-fronted goose	*Anser albifrons*	161	8	-	-
Black-headed gull	*Chroicocephalus ridibundus*	6	-	-	-
Pied avocet	*Recurvirostra avosetta*	3	-	-	-
Whooper swan	*Cygnus cygnus*	50	1	-	-
Mute swan	*Cygnus olor*	2	1	-	-
Black-necked drebe	*Podiceps nigricollis*	12	1	-	-
Common greenshank	*Tringa nebularia*	1	-	-	-
Grey plover	*Pluvialis squatarola*	1	-	-	-
Water rail	*Rallus aquaticus*	1	-	-	-
Little gull	*Hydrocoloeus minuta*	1	-	-	-
Common blackbird	*Turdus merula*	1	-	-	-
Meadow pipit	*Anthus pratensis*	1	-	-	-
Eurasian wigeon	*Anas penelope*	1	-	-	-
Common pheasant	*Phasianus colchicus*	2	-	-	-
Great egret	*Ardea alba* (*Egretta alba*)	25	-	-	-
Grey heron	*Ardea cinerea*	25	-	-	-
Total		504	27		

**Table 5:** Mixed-species samples, tested for IAV (January 2026).

Sample Type	Quantity	IAV	H5	H7
Mixed flock fecal samples (Eurasian Wigeon + Eurasian Teal)	50	2	-	-
Mixed heron flock samples (Great Egret + Grey Heron)	50	1	-	-
Total	100	3		

**Table 6:** Summary of sampling and testing.

Parameter	Species-Specific Samples	Mixed Faeces Samples	Total
Total samples collected	504	100	604
IAV-positive samples	27	3	30
H5-positive samples	0	0	0
H7-positive samples	0	0	0
Overall IAV positivity	5.36%	3.0%	4.97%

## Data Availability

Sequences mentioned in this study were deposited in GenBank using BankIT system for Influenzaviruses with accession numbers: H1 (PZ558585.1, PZ558541.1, PZ558332.1), H6 (PZ558586.1), and H4 (PZ559198.1 and PZ558750.1).

## Abbreviations

The following abbreviations are used in this manuscript:

HA	Hemagglutinin
LLC	Limited Liability Company
LPAIV	low-pathogenic avian influenza A viruses
ML	Maximum Likelihood
NA	neuraminidase
NDV	Newcastle disease virus
PSI	Private scientific institution
